# Regenerative Endodontics Maintained Arrest of External Inflammatory Root Resorption After Secondary Concussion Injury: A Case Report

**DOI:** 10.1155/crid/5571369

**Published:** 2026-06-08

**Authors:** Yilin Wang, Tianfeng Ouyang, Zeli Ma, Xin Li, Shanshan Chen, Ting Chen

**Affiliations:** ^1^ Department of Stomatology, Nanfang Hospital, Southern Medical University, Guangzhou, Guangdong, China, fimmu.com; ^2^ Department of Stomatology, Ganzhou People’s Hospital, Ganzhou, Jiangxi, China, gzsrmyy.com

**Keywords:** case report, pulpotomy, regenerative endodontics, root resorption, tooth injuries

## Abstract

This report highlights the unique occurrence of secondary trauma to a tooth previously treated with regenerative endodontic therapy (RET) and the successful outcome following this secondary injury. A 10‐year‐old girl sustained initial trauma to her maxillary central incisors. Tooth 11 sustained a complicated crown fracture and was treated with pulpotomy, which promoted complete root closure. Tooth 21 initially presented with an uncomplicated crown fracture; however, subsequent radiographic examinations revealed external inflammatory root resorption (EIRR) and periapical periodontitis. RET was performed to halt EIRR, and follow‐up radiographs confirmed complete healing, indicated by a well‐defined border around the resorption surface and the absence of apical radiolucency. Seven months after the initial trauma, the patient experienced a second injury, resulting in the concussion of both Teeth 11 and 21. No intervention was needed, but the teeth were closely monitored clinically. At the 26‐month follow‐up, both teeth remained asymptomatic with no pathological findings. This case demonstrates that RET effectively arrests EIRR in a short timeframe and maintains tooth stability even after subsequent mild trauma.

## 1. Introduction

Managing traumatized teeth is inherently difficult, which has a poorly predictable prognosis that depends on pulp vitality and treatment efficacy [1]. Inadequate management or delayed intervention can lead to serious complications, such as a loss of structural integrity or periodontal damage. One of the most severe complications is external inflammatory root resorption (EIRR), which can rapidly result in root destruction and tooth loss [2]. Conventional treatment for EIRR involves extended intracanal application of calcium hydroxide, initially for 3 weeks and then replaced every 3 months until resorption halts [1, 3]. However, this method presents several limitations, including low patient compliance, prolonged and unpredictable treatment duration, and an elevated risk of root fracture [4, 5]. Emerging studies indicate that regenerative endodontic treatment (RET) can effectively arrest and even reverse EIRR in a shorter timeframe [6], offering encouraging outcomes. RET promotes regeneration of pulp‐like tissue by inducing apical bleeding to recruit mesenchymal stem cells into the root canal, utilizing the blood clot as a natural scaffold under the regulation of growth factors. Nevertheless, current clinical evidence remains insufficient to endorse RET as a standard approach in dental practice [6–8].

Despite RET′s emerging role in trauma management, little is known about its response in cases where teeth are subjected to subsequent injury. Recurrent dental trauma presents a significant challenge, especially in teeth that have previously undergone endodontic treatment. Secondary injuries can compromise earlier interventions and negatively impact the long‐term prognosis of these teeth [9, 10]. Although the long‐term outcomes of endodontically treated permanent teeth following initial trauma are well established, limited evidence exists regarding their response to subsequent trauma. This case aims to illustrate the therapeutic efficacy of RET in managing EIRR and to evaluate the long‐term prognosis of teeth previously treated with pulpotomy and RET after subsequent trauma.

## 2. Case Report

A 10‐year‐old girl presented to the Pediatric Dentistry Department of Nanfang Hospital, Southern Medical University, reporting trauma to her maxillary anterior teeth sustained 8 days prior. She had previously received splinting for her maxillary anterior teeth at a local hospital immediately after the initial injury but sought further care at our institution because of persistent pain in the right upper central incisor. Her medical history was unremarkable. Because of the acute nature of the presentation, immediate treatment was prioritized over extensive photographic documentation; however, the key clinical findings are described in detail below.

Clinical examination revealed the following injuries, classified according to the International Association of Dental Traumatology (IADT) guidelines [1]:

• Tooth 11 presented with a complicated crown fracture (enamel–dentin fracture with pulp exposure). This was evidenced by visible enamel and dentin loss with clear pulp exposure, tenderness to percussion, patient‐reported persistent pain, normal mobility, and no associated swelling or sinus tract.
• Tooth 21 presented with an uncomplicated crown fracture (enamel–dentin fracture without pulp exposure). This was evidenced by visible enamel and dentin loss without pulp exposure, absence of bleeding from the fracture site, no tenderness to percussion, normal mobility, and no swelling or sinus tract.

Radiographic examination showed that both Teeth 11 and 21 were at Nolla stage 9, indicating nearly complete root development with open apices, a normal periodontal ligament space, and no evidence of root fracture, alveolar bone abnormality, periapical pathology, or a concomitant luxation injury (Figure 1a). The key treatment stages and follow‐up assessments are summarized in Table 1.

**Figure 1:**
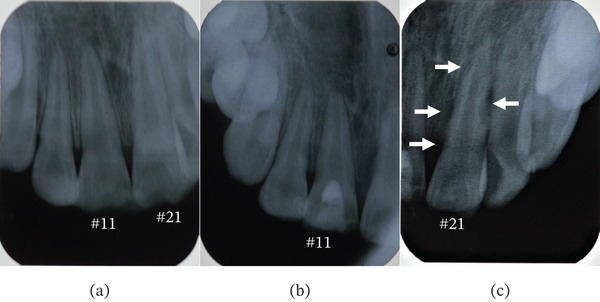
(a) Initial radiograph of Teeth 11 and 21. (b) One‐month follow‐up of Tooth 11 after pulpotomy. (c) One‐month follow‐up of Tooth 21 without intervention; white arrows indicate areas of root resorption.

**Table 1:** Treatment and follow‐up timeline for Teeth 11 and 21.

Time point	Tooth 11		Tooth 21	
	Intervention	Radiographic examinations	Intervention	Radiographic examinations
Initial trauma	Splinting	Not available	Splinting	Not available
Initial visit	Pulpotomy	Open apices, no periapical pathology	Fracture surface sealing	Open apices, no periapical pathology
1‐month FU	Monitoring	Normal	RET refused	Signs of EIRR
3‐month FU	Monitoring	Calcific barrier	RET initiated	Apical radiolucency, progressed EIRR
3 months + 3 weeks	Monitoring	Not available	RET completed	Not available
7‐month FU (second trauma)	Monitoring	Normal	Monitoring	Reduction in radiolucency
10‐month FU	Monitoring	Closed apex	Monitoring	Reduction in radiolucency
26‐month FU	Monitoring	Normal	Monitoring	Normal PDL space

Abbreviations: EIRR, external inflammatory root resorption; FU, follow‐up; PDL space, periodontal ligament space; RET, regenerative endodontic therapy.

### 2.1. Full pulpotomy for Tooth 11 With Complicated Crown Fracture

The patient′s parents were informed of the dental injuries and the available treatment options, and they provided informed consent for the proposed plan, which included pulpotomy for Tooth 11 and temporary dentin protection for Tooth 21, followed by observation. Local anesthesia was administered using 4% articaine with 1:100,000 epinephrine (Acteon, France). The teeth were disinfected and isolated with a rubber dam to maintain a sterile field. For Tooth 21, the fractured crown surface was sealed with Fuji IX glass ionomer cement (GC, Japan) to protect the dentinal tubules. For Tooth 11, the entire coronal pulp tissue was amputated to the level of the root canal orifices using a sterile diamond bur with a high‐speed handpiece under continuous water irrigation. The pulp stumps were irrigated with 5 mL of 1% sodium hypochlorite. Hemostasis was achieved with a 1% sodium hypochlorite–soaked cotton pellet applied for 15 s, followed by saline irrigation. No active bleeding was noted, indicating that healthy dental pulp had been reached. A 2‐mm layer of iRoot BP Plus (Innovative Bioceramix, Canada) was placed over the pulp stumps and adjusted with a moist cotton pellet to ensure adequate coverage. The iRoot BP Plus was then sealed with glass ionomer cement (GC, Japan) for long‐term protection. Postoperative instructions were given to the patient′s parents, and the patient was discharged.

### 2.2. RET for Tooth 21 With EIRR and Apical Periodontitis

At the 1‐month follow‐up, both teeth were asymptomatic. Tooth 11 exhibited no tenderness to percussion or palpation, responded positively to cold testing, and showed no mobility. Tooth 21 also showed no tenderness or mobility but had negative responses to both electric and thermal pulp sensibility tests. Periapical radiographs revealed no radiolucent lesions around Tooth 11 (Figure 1b), whereas Tooth 21 displayed signs of EIRR (Figure 1c). Tooth 21 was diagnosed with pulp necrosis. RET was recommended for Tooth 21; however, the patient′s parents opted to postpone treatment because of the absence of symptoms.

At the 3‐month follow‐up, Tooth 11 was asymptomatic with normal vitality, color, and mobility. Periapical radiographs revealed no radiolucency, with evidence of thickened root canal walls and a continuous, high‐density calcific barrier beneath the pulp capping material (Figure 2a). In contrast, Tooth 21 exhibited crown discoloration, sensitivity to percussion, and Class I mobility. Cold and electric pulp sensibility tests were negative. Radiographic evaluation revealed periapical radiolucency and progression of EIRR (Figure 2a). A diagnosis of pulp necrosis with chronic apical periodontitis was made for Tooth 21. RET was recommended, and the patient′s parents agreed to proceed with treatment due to the rapid progression of the disease.

**Figure 2:**
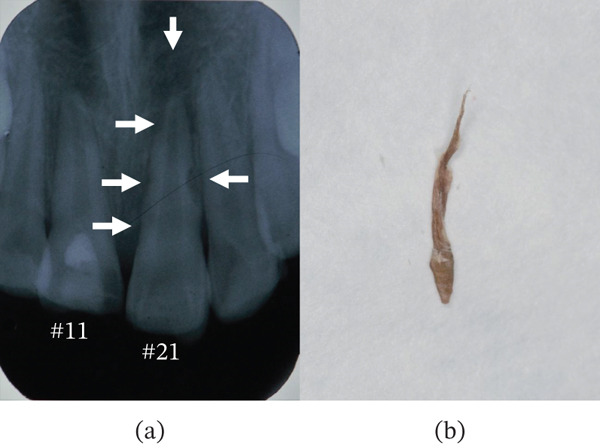
(a) Three‐month follow‐up radiograph of Tooth 11 after pulpotomy and Tooth 21 without intervention; white arrows indicate areas of root resorption and periapical radiolucency. (b) Necrotic pulp tissue from Tooth 21.

Under local anesthesia and rubber dam isolation, endodontic access was achieved, and the necrotic pulp was removed entirely (Figure 2b). The working length was determined radiographically as 21 mm (file positioned 1 mm short of the apex). A size #25 K‐file was used to gently “brush” the canal walls circumferentially with minimal dentin removal. The root canal was irrigated with 20 mL of 1.5% sodium hypochlorite for 5 min (needle positioned 2 mm from the apex at 19 mm, activated ultrasonically), followed by 5 mL of sterile saline. After drying with sterile paper points, the canal was medicated with calcium hydroxide paste (Ivoclar Vivadent AG, Liechtenstein) to promote healing and reduce infection and sealed with glass ionomer cement (GC, Japan). At the second appointment 3 weeks later, the patient remained asymptomatic with no signs of inflammation. Under local anesthesia and rubber dam isolation, the access cavity was reopened. The canal was irrigated with 20 mL of 17% ethylenediaminetetraacetic acid (Pulpdent, USA) for 5 min (ultrasonically activated), followed by 5 mL of sterile saline to release bioactive molecules from the dentin matrix. Bleeding was induced using a size #25 K‐file extended 2 mm beyond the apical foramen (23 mm), achieving a blood clot filled to 2 mm below the cementoenamel junction. The blood clot serves as a natural fibrin‐based scaffold that is rich in platelets, mesenchymal stem cells, and growth factors, which supports tissue regeneration and healing. A layer of iRoot BP Plus (Innovative Bioceramix, Canada) was placed over the blood clot for its excellent sealing ability and aesthetic properties, and the access cavity was sealed with glass ionomer cement (GC, Japan). Postoperative instructions were provided to the patient′s parents, and the patient was discharged.

### 2.3. Recurrent Trauma to Teeth 11 and 21 After Treatment

At the 7‐month follow‐up, the patient reported a history of recurrent trauma to the maxillary anterior teeth (Figure 3). Clinical and radiographic examination of Teeth 11 and 21 revealed the following findings:

• Tooth 11: No coronal discoloration. Restoration margins remained intact. Tender to percussion, no mobility, and normal responses to cold and electric pulp sensibility tests. Radiographic examination showed no abnormalities (Figure 4a).
• Tooth 21: Notable coronal discoloration (consistent with prior treatment sequelae). Restoration margins remained intact. Tender to percussion, no mobility, and cold and electric pulp sensibility tests within normal limits. Radiographs demonstrated a reduction in the previously noted periapical and EIRR radiolucency (Figure 4a).

**Figure 3:**
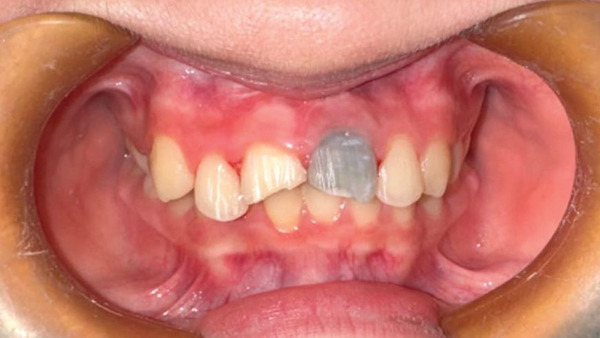
Intraoral photograph showing the second concussion injury to Teeth 11 and 21 following treatment. Coronal discoloration is evident in Tooth 21.

**Figure 4:**
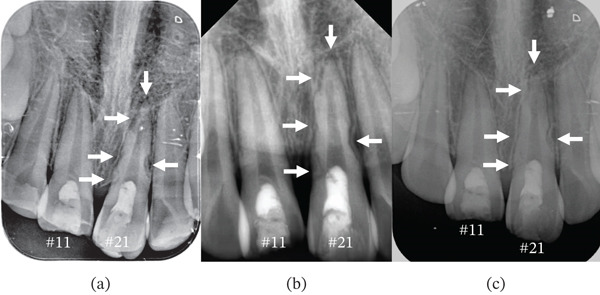
(a) Seven‐month follow‐up radiograph. (b) Ten‐month follow‐up radiograph. (c) Twenty‐six‐month follow‐up radiograph. White arrows indicate the healed areas of root resorption and periapical inflammation, which are surrounded by a sclerotic border.

The IADT 2020 Guidelines do not define a special subcategory for secondary trauma in previously treated teeth [1]. Such events are evaluated as new injury episodes and classified based on current clinical and radiographic findings. In this case, the presence of percussion tenderness combined with the absence of mobility, displacement, or new radiographic abnormalities means the presentation fulfills the diagnostic criteria for concussion. Because the teeth had been treated, the prognosis following further trauma was unclear. Management consisted of continued clinical and radiographic monitoring of both teeth.

At both the 10‐month and 26‐month follow‐up evaluations, the clinical findings remained consistent with those of previous assessments. Radiographic examination of Tooth 11 revealed a well‐defined calcified bridge beneath the pulp capping material, complete root maturation with a closed apical foramen, and no signs of periapical pathology. In Tooth 21, the previously noted apical radiolucency had fully resolved. The root surfaces showed evidence of remodeling and were bordered by a thin radiolucent line consistent with the periodontal ligament space. The root canal wall had thickened, with calcification observed in the apical third (Figure 4b,c).

## 3. Discussion

This case report describes the successful management of EIRR using RET in a young permanent tooth and presents a unique observation of the treated tooth′s response to subsequent mild trauma. At the 7‐month follow‐up, periodontal tissue remodeling was evident, and pulp sensibility tests remained within normal limits. These outcomes underscore the advantages of RET over conventional methods, including a shorter treatment duration, enhanced patient compliance, thickened root canal walls, and continued root development [11]. Recent studies also support RET as a promising strategy for EIRR [12–15].

Although the reestablishment of pulp sensibility does not necessarily indicate true pulp regeneration, it suggests the presence of functional neural elements within the newly formed tissue. Recent meta‐analyses and reviews report that approximately 50%–56% of RET cases exhibit return of pulp sensibility [16]. Histologic studies and expert consensus indicate that the tissue formed within the root canal after RET typically comprises a mixture of bone‐, dentin‐, cementum‐, and periodontal ligament‐like tissues and fibrous connective tissue, rather than fully organized pulp tissue. This reparative tissue can nonetheless maintain tooth function, enable perception of external stimuli through innervation‐like responses, and provide an immune‐inflammatory defense against microbial invasion [17].

A notable feature of this case is the 2‐month delay between EIRR diagnosis and RET initiation, due to parental deferral. The pathological mechanism of EIRR underscores why timely intervention is essential. Trauma or pulp necrosis damages the root cementum layer, exposing dentinal tubules and creating pathways for bacteria and their toxins. Bacterial products stimulate periodontal ligament fibroblasts to release pro‐inflammatory factors, including RANKL and M‐CSF, which activate odontoclast and osteoclast precursors, leading to progressive destruction of the root and surrounding bone, respectively [18, 19]. Delayed treatment prolongs infection and inflammation, potentially expanding the resorption area, compromising periodontal ligament integrity, and worsening long‐term prognosis.

Despite these promising outcomes, the choice of RET must account for its potential complications, primarily root canal calcification and crown discoloration [20]. Apical calcification, possibly due to the deposition of reparative dentin or the ingrowth of adjacent tissues into the canal space [21], does not typically impede healing but may complicate future endodontic access. Song et al. reported that 62.1% of cases using a blood clot scaffold exhibited intracanal calcification [22]. Aesthetic concerns are paramount for anterior teeth, with crown discoloration reported in 44%–62% of RET cases [23]. The European Society of Endodontology recommends using Ca (OH)_2_ instead of antibiotic pastes to minimize risks of antibiotic resistance and tooth discoloration [24]. Evidence suggests that PRF may cause less discoloration than blood clots [25], and intracoronal bleaching offers a viable corrective option [26].

The long‐term prognosis of immature permanent teeth treated with pulpotomy or RET has been well documented. However, limited data exist regarding their response to subsequent traumatic events [10]. Secondary trauma can result in substantial injury to pulpal or periodontal tissues, potentially triggering inflammation or infection and increasing the risk of complications [9, 10]. Prognosis may depend on trauma severity, pulpal status, and the nature of prior treatment. Prado et al. [27] successfully managed a RET‐treated tooth with pulp chamber re‐exposure after new trauma using an alternative obturation technique. Cehreli et al. [28] observed that among three RET‐treated teeth that sustained subsequent luxation injuries, one progressed to require root canal treatment, whereas the other two remained asymptomatic for over a decade.

In this case, the RET‐treated tooth and adjacent pulpotomy‐treated teeth responded favorably to mild concussion, showing no pathology at 10‐ and 26‐month follow‐ups. Several mechanisms may explain this favorable response. In teeth treated with pulpotomy, the calcific bridge likely enhances the coronal seal and increases the distance external stimuli must travel to reach the vital pulp, whereas the retained pulp tissue maintains an adaptive capacity comparable to that of healthy pulp. In teeth treated with RET, the thickened root walls further bolster fracture resistance, contributing to the tooth′s long‐term survival. However, the biological mechanisms underlying the response of the regenerated intracanal tissue to secondary injury remain largely unexplored, and further research is needed to elucidate how the composition of reparative tissue influences its behavior under traumatic conditions.

Limitations of this report include the absence of CBCT imaging (due to ethical radiation concerns in pediatrics), nonstandardized radiographic angulation, the single‐case nature (precluding generalizability), and relatively short follow‐up for long‐term assessment such as calcification progression or reinfection risk. Further longitudinal studies with larger cohorts and standardized imaging protocols are needed to better understand the biological behavior of teeth following repeated trauma.

## 4. Conclusion

This case demonstrates that RET effectively arrests EIRR in a short timeframe and maintains tooth stability even after subsequent mild trauma. Clinicians should consider RET as a viable treatment option for EIRR in immature permanent teeth, and close posttraumatic monitoring is recommended even after successful regenerative procedures.

## Author Contributions

**Yilin Wang:** visualization, writing—original draft. **Tianfeng Ouyang:** methodology, validation. **Zeli Ma:** investigation. **Xin Li:** data curation. **Shanshan Chen:** supervision. Ting Chen: conceptualization, writing—review and editing. Yilin Wang and Tianfeng Ouyang contributed equally to this work.

## Funding

No funding was received for this manuscript.

## Disclosure

All authors have read and approved the final version of this manuscript. Each author has contributed significantly to the work and agrees to be personally accountable for their own contributions and for ensuring that questions regarding the accuracy or integrity of any part of the work are appropriately addressed.

## Ethics Statement

Ethical approval was not required for this single case report. Written informed consent for treatment and publication was obtained from the patient′s legal guardian. The study was conducted in accordance with the principles of the Declaration of Helsinki.

## Consent

Written consent for publication of the clinical and radiographic details was obtained from the patient′s parent.

## Conflicts of Interest

The authors declare no conflicts of interest.

## Data Availability

The data that support the findings of this study are available on request from the corresponding author. The data are not publicly available due to privacy or ethical restrictions.
